# Note on the Difference between the Principal Balance Analysis with *NearestBalance* and Constrained Methods

**DOI:** 10.1128/msystems.00164-23

**Published:** 2023-03-13

**Authors:** Vera E. Odintsova, Natalia Klimenko, Alexander V. Tyakht

**Affiliations:** a Atlas Biomed Group - Knomx LLC, Moscow, Russia; b Center for Precision Genome Editing and Genetic Technologies for Biomedicine, Institute of Gene Biology, Russian Academy of Sciences, Moscow, Russia; University of California San Diego

**Keywords:** compositional analysis, microbiome, principal balance analysis

## LETTER

This letter is about a particular case of approximation of a compositional vector by the nearest balance—its application to principal balance analysis (PBA). It compares two methods: our *NearestBalance* approach (1) and the constrained method suggested previously in references 2 and 3. We recognize that they have the same underlying idea, and we apologize for having missed this fact in our original paper. Still, due to algorithmic details the constrained method provides a suboptimal solution, while the *NearestBalance* approach guarantees the minimization of the approximation error.

This letter was motivated by a discussion at the conference on compositional data analysis CoDaWork2022. We presented our nearest balance approach to approximation of a compositional vector (1) and mentioned its application to principal balance analysis (PBA). J. A. Martin-Fernández asked what the difference is between this method and the constrained algorithm suggested in reference 2 and applied to PBA in reference 3. Indeed, both of them state that they approximate the principal components by the nearest balances. Here, we would like to clarify the difference—by emphasizing that the authors of reference 3 were the first to suggest the idea itself but showing that the algorithm they used is suboptimal, while ours (1) provides the exact solution.

As a first step, we made note of the fact that results of the methods do not coincide, though they solve exactly the same problem. Our paper (1) contains application of the algorithms to a Crohn’s disease data set (4). Implementation of the constrained method is taken from the *coda.base* package, and that of the other one is from the *NearestBalance* package. The algorithms provided a slightly different explanation of the variance by the first principal balance (25% by *NearestBalance* versus 24.71% by the constrained method). We extended the comparison to check whether there was a difference in the balances themselves and their angle to the first principal component (PC1). It was actually present: the number of taxa included in balances is different (*n*_NB_ = 27 versus *n*_C_ = 29), and the *NearestBalance* provides a smaller angle to PC1 (α_NB_ = 25.14° versus α_C_ = 26.14°). As both methods target minimizing this angle, the constrained algorithm does not exactly reach the goal.

The difference in results is explained by the differences in the algorithms themselves. In brief, both of them are based on expression of the angle cosine:

$$\text{cos}\left(\text{α}\right)=\frac{1}{\left\|\hat{v}\right\|}\sqrt{\frac{\mathrm{rs}}{r+s}}\left\{\frac{1}{r}\left[{\hat{v}}_{1}^{+}+\;\ldots \;+{\hat{v}}_{r}^{+}\right]\;-\;\frac{1}{s}\left[{\hat{v}}_{1}^{-}+\;\ldots \;+{\hat{v}}_{s}^{-}\right]\right\}$$

where *r* and *s* are the number of parts in the numerator and denominator of the balance, ${\hat{v}}_{i}^{+}$ (i = 1, …, *r*) and ${\hat{v}}_{j}^{-}$ (j = 1, …, *s*) are clr-components of the vector (PC1) related to them, and ‖·‖ denotes the Euclidean norm which can be removed from the objective function. The *NearestBalance* algorithm searches through all possible sizes of *r* and *s* of the two groups of parts included in the balance; on each step, the *r* and *s* are fixed, and thus, the cosine is maximized by including *r* parts with maximal clr-components of PC1 in one group and *s* parts with minimum ones in the other group. The constrained algorithm searches through all possible total numbers of parts in a balance, i.e., through values of *n* = *r* + *s* from 2 to *D*. At each step the parts are sequentially included in the balance in the order of their absolute values of PC1 clr-components, and they are related to the numerator or denominator with respect to the sign of the components. The only exception is the first step, when the two-component composition is constructed of the parts with the highest and the lowest clr-components of PC1 whichever absolute values they have. Both methods calculate the cosine at each step of their search, and then the optimal number of parts is selected. The main difference is that the constraint algorithm does not compare all variants of *r* and *s* that sum to fixed *n*; it takes the balance obtained on the previous step (*r* + *s *= *n* − 1) and adds the part of composition with the highest absolute value of the approximated vector’s clr-component.

**FIG 1:**
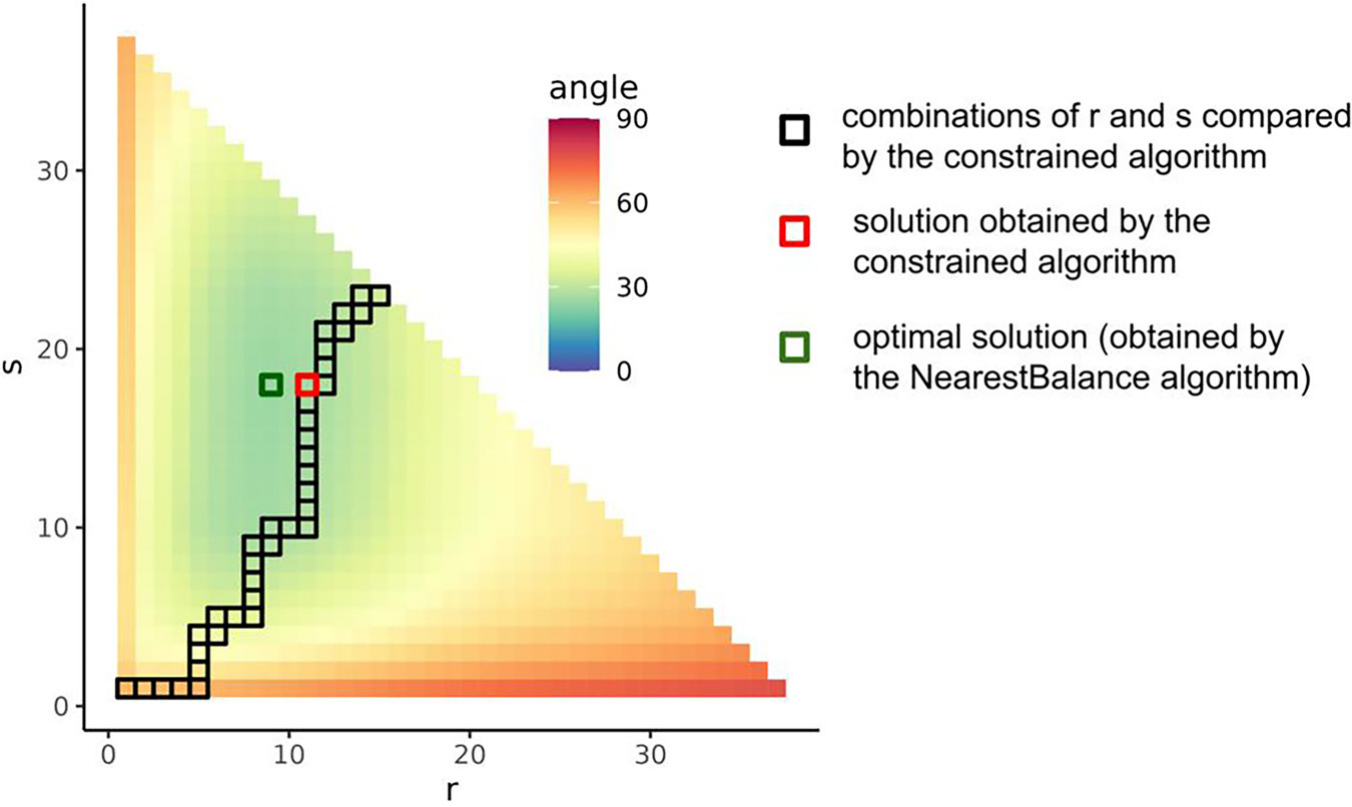
Difference in the number of *r* and *s* combinations evaluated in the *NearestBalance* and constrained algorithms: *NearestBalance* compares cosine values for all possible *r* and *s* combinations, and the constrained algorithm compares only the ones marked by the black frame.

Thus, the constrained algorithm searches through a substantially smaller subset of balances. Figure 1 illustrates the difference on the Crohn’s disease data set. The incompleteness of the search is the source of suboptimality of the constrained method: it does not find the optimal solution because it does not include the appropriate (*r*, *s*) pair in the comparison.

On the other hand, this incompleteness makes the constrained algorithm substantially faster. It needs *D* − 1 steps for a *D*-part composition; the complexity of *NearestBalance* is proportional to (*D* − 1)^2^.

To sum it up, the two algorithms—*NearestBalance* and constrained—aim to find the nearest balance to a compositional vector. Only the *NearestBalance* actually provides it, while the constrained method finds a suboptimal approximation. However, in practice the constrained algorithm may yield a result quite similar to the optimal one, it is faster (especially for high-dimensional compositions), and additionally it creates a complete orthonormal log-ratio basis, while the *NearestBalance* in its current implementation provides only the two first principal balances.

Noteworthy, the authors of reference 3 were the first ones who suggested using the nearest balance for PBA. We apologize for not acknowledging this fact in reference 1. Our paper (1) suggests an algorithm which finds exactly the nearest balance, suggests a wider use to the approach, and provides grounds for a special case of combination with regression analysis.

Function *find_nearest_balance_clr()* from the *NearestBalance* R package was used for approximation of the first principal balance by the algorithm described in reference 1. Function pb_basis() with the ‘constrained’ method from the *coda.base* package was used for the constrained PBA. The constrained algorithm was additionally implemented as a standalone function which takes a CLR-vector as an input, because the *coda.base* package contains only its application to PBA. We ensured that for the Crohn’s disease data set, the new function returns exactly the same result as *pb_basis()*.

The comparison code is available at https://bitbucket.org/knomics/nearest_balance_for_paper.
